# Alveolar ridge resorption after tooth extraction: A consequence of a fundamental principle of bone physiology

**DOI:** 10.1177/1758736012456543

**Published:** 2012-08-16

**Authors:** Stig Hansson, Anders Halldin

**Affiliations:** 1 Astra Tech AB, Mölndal, Sweden; 2 Department of Prosthodontics, Faculty of Odontology, Malmö University, Malmö, Sweden

**Keywords:** bone resorption, tooth extraction, implant

## Abstract

It is well established that tooth extraction is followed by a reduction of the buccolingual as well as the apicocoronal dimension of the alveolar ridge. Different measures have been taken to avoid this bone modelling process, such as immediate implant placement and bone grafting, but in most cases with disappointing results. One fundamental principle of bone physiology is the adaptation of bone mass and bone structure to the levels and frequencies of strain. In the present article, it is shown that the reduction of the alveolar ridge dimensions after tooth extraction is a natural consequence of this physiological principle.

It is well established that tooth extraction is followed by a reduction of the buccolingual as well as apicocoronal dimension of the alveolar ridge at the edentulous site.^1,2^ It has been suggested that immediate implant placement into fresh extraction sockets might counteract this catabolic process and preserve the dimensions of the alveolar ridge.^3–6^ However, studies in humans^7^ and experiments in dogs^8,9^ have belied this hypothesis. In another dog study, it was found that the resorption of the buccal/lingual walls occurred in two overlapping phases. In a first phase, the bundle bone was resorbed and replaced with woven bone. The second phase included resorption from the outer surface of both bone walls.^10^ It was stated that the reason for this additional bone loss was not understood. In a dog study, extraction sockets were found to be filled by woven bone after 1 month and after 3 months a cortical ridge including woven and lamellar bone had been formed.^11^ After 6 months, woven bone was being replaced with lamellar bone and bone marrow. The application of freeze-dried bone allograft in combination with a membrane was found to improve the ridge dimensions in patients after 6 months compared to a control, both vertically and horizontally.^12^ In studies in dogs, grafting with Bio-Oss™ collagen in extraction sockets improved the ridge dimensions after 6 months compared to a control,^13^ while grafting with autologous bone did not.^14^

The above literature gives the impression that the reason for bone loss after tooth extraction is unknown. In the year 1881, Roux^15^ suggested that the loss of alveolar bone occurring after tooth loss in the old age is an example of disuse atrophy. His reasoning was that after tooth loss, the forces on the bone are reduced, which means that less bone is needed and that the body gets rid of bone that is not sufficiently used. Our knowledge of bone physiology has expanded greatly since 1881.

Wolff’s^16^ law suggests that bone tissue adapts its mass and structure to the mechanical demands. A more detailed discussion on this subject requires some insights in the discipline mechanics of materials. When a structure, for example, the mandible, is loaded, it is deformed. There are stresses and strains in the structure. An infinitesimal cubic element in the structure is considered (Figure 1(a)). The stresses on the surfaces of this cube are expressed: tensile/compressive stresses perpendicularly to the surfaces and shear stresses parallel to the surfaces. The cube can be rotated, so that the shear stresses disappear and we only have stresses perpendicularly to the surfaces (Figure 1(b)). These latter stresses are called principal stresses. The corresponding strains are called principal strains (Figure 1(c)). With knowledge of the geometry of a structure, the material properties and the loads upon the structure the stresses and strains can be calculated.^17^ Figure 2 illustrates the concept of strain. Figure 2(a) shows a piece of material that is unloaded. In Figure 2(b), it is subjected to a tensile force. The piece is elongated. The elongation (ΔL) divided by the original length (L) gives the strain (ΔL/L). In this case, the strain is positive. With a compressive load, the piece of material becomes shorter. The strain is negative. Strains in, for example, cortical bone are small. For this reason, the unit microstrain is often used. If the elongation is 1% of the original length, the strain is 10,000 microstrain.

**Figure 1.**
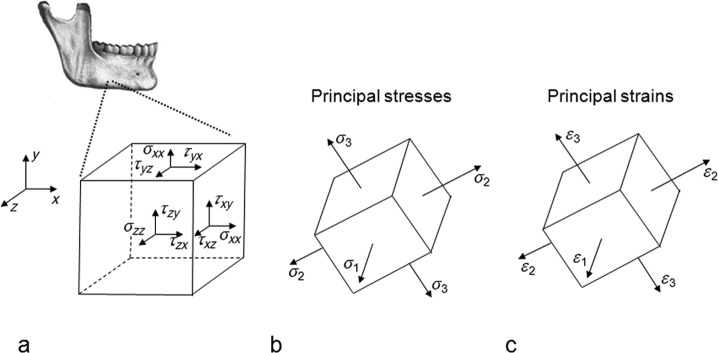
(a) Tensile/compressive stresses and shear stresses on the surfaces of an infinitesimal cubic element, (b) principal stresses and (c) principal strains.

**Figure 2.**
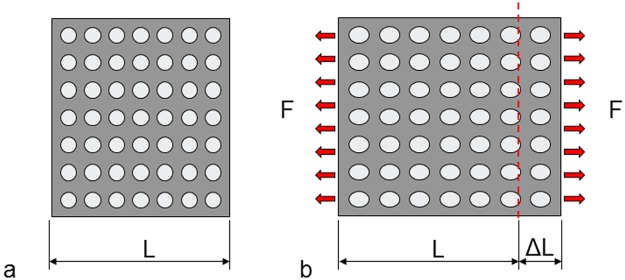
(a) A piece of a material that is unloaded and (b) a distributed tensile force (F) elongates the piece of material.

There is a wealth of literature testifying of profound effects of strain on bone mass and bone structure. The lamellae of cancellous bone are preferentially aligned with the principal strains caused by the dominating loads.^16,18,19^ This enables the most economical use of the bone material. Changes in loading direction result in changes in the directions of the principal strains, and the lamellae of the cancellous bone realign with the new principal strain directions.^20^ Petrtýl et al.^21^ found that the Haversian systems of cortical bone preferentially are aligned with the first principal strain caused by the dominating loads. This also means an economical use of the bone material. The bone mass primarily depends on the magnitude of the strains^22^ and the number of strain cycles per time unit.^23^ Based on a compilation of animal experimental data, Qin et al.^23^ proposed the following formula for a daily stress stimulus,

(1) $$\Psi_{b}={\left(\sum_{\text{day}}n_{i}\sigma_{i}^{m}\right)}^{1/m}$$

where *n*_*i*_ is the daily number of cycles of loading type *i, σ*_*i*_ is the stress associated with loading type *i* and the exponent *m* is a constant, the value of which depends on the daily number of loading cycles. By substituting *σ*_*i*_ in equation (1) with *ϵ*_*i*_/*E*, where *ϵ*_*i*_ is the strain associated with loading type *i* and *E* is the modulus of elasticity of the bone, a formula for a daily strain stimulus is obtained (equation (2))

(2) $$\Psi_{b}={\left(\sum_{\text{day}}n_{i}{\left(\varepsilon_{i}/E\right)}^{m}\right)}^{1/m}$$

Qin et al.^23^ found that the strain stimulus needed per day to maintain bone mass could be expressed by the following formula

(3) $$y=10^{2.28}{\left(5.6-\log_{10}x\right)}^{1.5}$$

where *x* is the number of loading cycles per day and *y* is the strain magnitude. Rubin et al.^24^ observed that the maximum bone strains measured in the metacarpal bone of a galloping horse, the tibia of a running human, the femur of a running sheep, the humerus of a flying goose and the mandible of a chewing macaque are remarkably similar, ranging between 2000 and 3500 microstrain. These strains are about 50% of the yield strain of cortical bone, indicating that nature applies a safety factor of approximately 2 when designing bones. The aim of the present study was to investigate whether the observed changes in alveolar ridge dimensions, after tooth extraction, can be understood within the framework of established principles of bone physiology.

## Methods and results

### Bending of a beam

**Figure 3.**
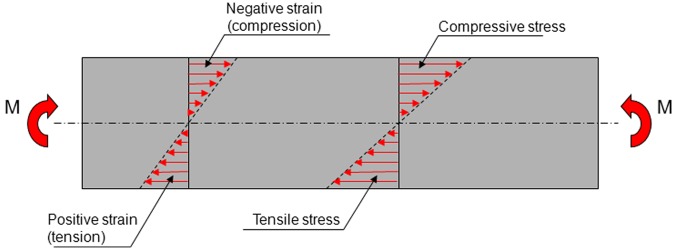
A beam, with a symmetric cross section, subjected to pure bending. The bending moment (M) induces tensile stresses and strains in the lower half of the beam and compressive stresses and strains in the upper half.

Consider the beam in Figure 3 that is subjected to pure bending. The beam is assumed to have a symmetrical cross section. The bending moment (M) gives rise to stresses and strains in the beam. At the longitudinal axis of the beam, the stresses and strains are zero. When the strains are below the yield strain of the material (6000 microstrain for cortical bone), there is a linear relationship between stress and strain. The stresses create an internal bending moment that exactly counterbalances the external bending moment (M).

### The mandible as a beam subjected to bending moments

Consider a mandibular tooth. When the tooth is loaded, it will induce a mechanical stimulation, strains, in the bone immediately adjacent to the tooth. The loading of more distant teeth, the action of the masticatory muscles and the reaction forces at the temporomandibular joints will give rise to bending moments in the mandible. These bending moments will also give rise to strains in the bone adjacent to the tooth in question. A steady-state condition is assumed to prevail, which means that the sum of these strains will represent the strain stimulus needed to maintain bone mass as proposed by Qin et al.^23^

**Figure 4.**
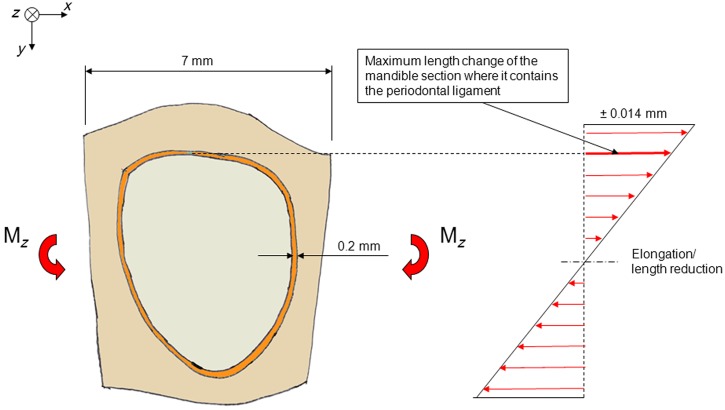
Schematic picture of a section of a mandible seen from above. The section contains a tooth, the periodontal ligament and the surrounding bone. The mandible section is subjected to bending in the horizontal plane. The tensile and compressive strains and the length changes are the highest buccally and lingually.

Consider a section of the mandible containing one tooth (Figure 4). The mandible section is assumed to be subjected to a bending moment (M_*z*_), which gives rise to deformations in the horizontal plane (vertical moment vector). Consider a bending moment of such a magnitude that an average strain of ±2000 microstrain arises in the buccal and lingual extremities of the mandible section. This is an unusually high strain for cortical bone.^24^ As the length of the mandible section is assumed to be 7 mm, these bending moments will give rise to a maximum elongation or reduction in the length of the section, which amounts to 0.002 × 7 = 0.014 mm. The length changes of the part of the mandible section that contains the periodontal ligament are smaller (Figure 4). Theoretically, these latter length changes will be absorbed by the bone, by the periodontal ligament and by the tooth. Subtracting the part that is absorbed by the bone, the maximum length changes that are absorbed by the periodontal ligament and the tooth will be well below 0.014 mm.

**Figure 5.**
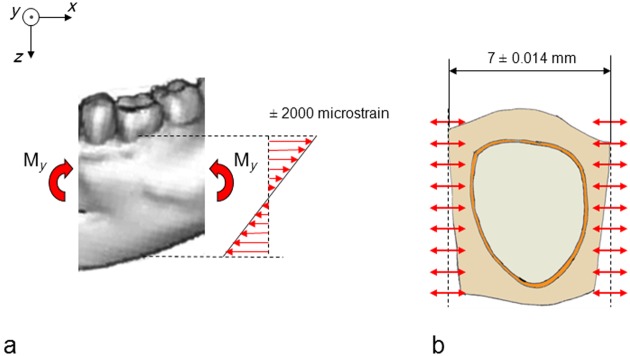
(a) A section of a mandible containing one tooth. The mandible section is subjected to bending in a vertical plane, which creates strains. The strains are highest in the upper and lower extremities of the section. (b) The mandible section seen from above. The length changes in the uppermost part are shown.

In Figure 5, the mandible section is assumed to be subjected to bending in vertical direction, which results in an average strain in the uppermost part of ±2000 microstrain. This implies that length changes amount to ±0.014 mm, which will be absorbed by the bone, the periodontal ligament and the tooth together. The maximum length changes that will be absorbed by the periodontal ligament and the tooth together will be below 0.014 mm.

### From a mechanical point of view, the mandible behaves as if the space occupied by the periodontal ligament and the tooth was empty

The thickness of the human periodontal ligament is about 0.1–0.3 mm. Assume an average thickness of 0.2 mm.^25^ Pini et al.^26^ derived stress–strain curves in compression and tension for bovine periodontal ligaments. With strains below ±10%, the stresses were close to 0. This finding was confirmed by Sanctuary et al.^27^ who investigated the mechanical properties of the bovine periodontal ligament. The stress–strain curves exhibited a central ‘zero zone’ in which the periodontal ligament behaved like a fluid. In this zone, straining of the periodontal ligament sample did not result in any significant stress response. Independently of strain rate, no significant stresses appeared below a strain of ±20%. The above discussed length change of less than 0.014 mm distributed over two periodontal ligament passages (2 × 0.2 = 0.4 mm) implies a strain that is less than 0.014/0.4 = 0.035. It can be concluded that the stresses in the periodontal ligament with this strain are negligible. This means that no stresses are transmitted from the bone to the tooth. The tooth does not participate in resisting the bending moments. From a mechanical point of view, the mandible behaves as if the space occupied by the periodontal ligament and the tooth was empty.

### When the extraction socked is filled with bone the mandible becomes stiffer and the strains are reduced

**Figure 6.**
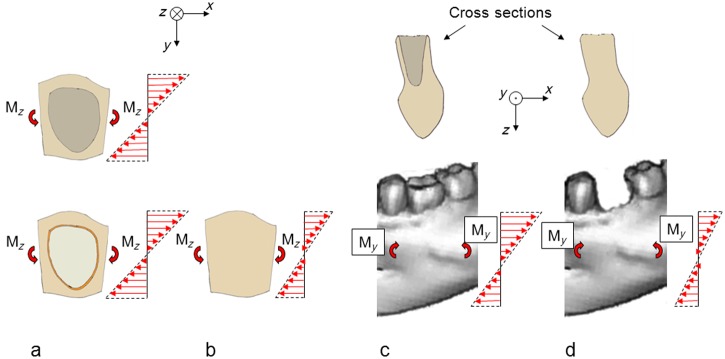
(a) A mandible section containing one tooth (bottom) or one extraction socket (top), as seen from above. The mandible section is subjected to bending in a horizontal plane with a specific bending moment (M_*z*_). The stiffness of the mandible section is the same in these two cases. Consequently, the strains are the same. (b) When the extraction socket is filled with bone, it becomes stiffer and the strains are reduced. (c) A mandible section containing one tooth (bottom) or one extraction socket (top). The mandible section is subjected to bending in a vertical plane with a specific bending moment (M_*y*_). The stiffness of the mandible section is the same in these two cases. Consequently, the strains are the same. (d) When the extraction socket is filled with bone, it becomes stiffer and the strains are reduced.

After resorption of the bundle bone, the extraction socket will gradually be filled with lamellar and cancellous bone (Figure 6), which will make the mandible section stiffer both with respect to horizontal bending and vertical bending. A consequence of this is that with unchanged bending moments, the bone strains will be reduced. The absence of the extracted tooth represents a further strain reduction. Reduced bone strains result in bone loss.^23,24^ In a study in dogs, the right forelimb was functionally isolated, by encasing in plaster, while the left forelimb served as control.^28^ Functional isolation results in reduction of the bone strains. After 40 weeks, approximately 50% of the bone mass was lost on the third metacarpal, 42% on the radius, 35% on the ulna and 28% on the humerus of the experimental limb. In total, 80%–90% of the bone loss occurred at the periosteal surface. Thus, bone resorption, mainly at the external bone envelope, resulting in reduced vertical and horizontal dimensions of the mandible, appears to be a natural consequence of tooth extraction. The bone resorption can be expected to continue until the bone strains have reached the levels of the pre-extraction time with healed conditions in the extraction socket.

### An implant will further increase the stiffness of the mandible

**Figure 7.**
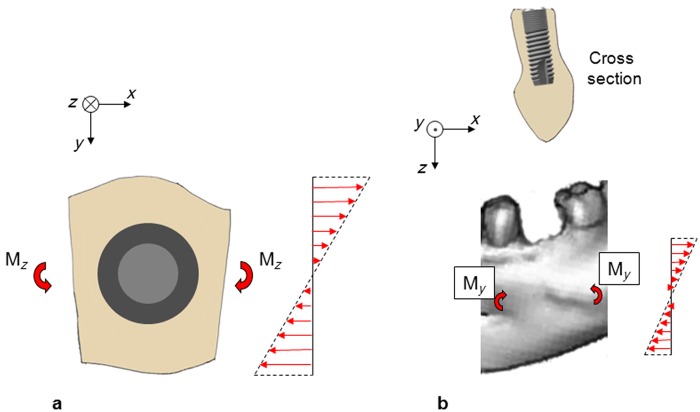
(a) A mandible section, containing an implant, as seen from above. The mandible section is subjected to bending in the horizontal plane. The implant makes the mandible section stiffer, and the strains are reduced. (b) A mandible section containing an implant. The mandible section is subjected to bending in a vertical plane. The implant makes the mandible section stiffer, and the strains are reduced.

Consider the mandible section, now containing an implant, that is subjected to a bending moment (M_*z*_), which gives rise to deformations in the horizontal plane (Figure 7(a)). About half of the implant will be subjected to compressive stresses, and the other half will be subjected to tensile stresses. On the compression side, the implant will contribute to the stiffening of the mandible. The magnitude of this stiffening effect depends on the implant design and the implant material. The modulus of elasticity of titanium, cortical bone and cancellous bone are about 107, 19 and 0.8 GPa, respectively.^29^ Since titanium is much stiffer than cortical and cancellous bone, the implant will, compared to the situation with bone completely filling the previous extraction socket, further increase the stiffness of the mandible on the compression side. On the tension side, the situation is more complicated. The tensile strength between implant and bone is limited.^30^ Theoretically, this tensile strength can locally be exceeded, and a small gap arise between implant and bone. This will have a reducing effect on the mandible stiffness. However, the net effect of the implant should be a further stiffening of the mandible as compared to the situation with bone filling the previous extraction socket. The same line of argument applies to the situation when the mandible section is subjected to vertical bending (Figure 7(b)). Thus, on theoretical grounds, immediate implant placement into fresh extraction sockets should not be expected to prevent the reduction of the buccolingual or apicocoronal dimensions of the alveolar ridge.

### Retention elements at the endosseous neck portion of the implant should preserve the apicocoronal dimension of the alveolar ridge

An increased resistance to bending of the mandible, both in horizontal and vertical directions, seems to be an inevitable consequence of the replacement of the tooth and periodontal ligament by an implant and bone. An increased resistance to bending implies reduced strains provided that the magnitude of the bending moments remains unchanged. Nature’s normal response to reduced bone strains is to reduce the bone mass and architecture in such a way that the daily stress/strain stimulus needed to maintain bone mass is reached again.^23^ This is normally done by resorption at the external bone envelope.^28^

The resistance to bending of the mandible in horizontal and vertical directions can be reduced by reducing the buccal-lingual dimensions, by reducing the apicocoronal dimensions or by doing both. Theoretical^31,32^ and clinical studies^33–35^ have demonstrated that the reduction of the apicocoronal dimensions can be reduced to a minimum if the endosseous neck portion is equipped with retention elements of suitable design. With such retention elements of a dental implant, the daily stress/strain stimulus needed to maintain bone mass seems to be reached for the coronal-most bone. Thus, with an implant that maintains the marginal bone level, nature achieves the required reduction in resistance to bending of the mandible primarily by reduction of the buccal-lingual dimensions.

### A theoretical possibility to also maintain the buccal-lingual dimensions of the mandible

The bending moment required to produce a certain strain on the surface of the beam in Figure 3 is proportional to the product of a geometric entity called *section modulus* and the *modulus of elasticity* of the material. For a beam with a circular cross section, the *section modulus* equals *πD*^3^/32, where *D* is the diameter of the cross section. The fact that the diameter, *D*, is raised to the power of three means that the *section modulus* is very sensitive to the size of the cross section. In the mandible containing one implant, there are three different materials: cortical bone, cancellous bone and the implant material. The modulus of elasticity of cortical bone is about 20–50 times as high as that of cancellous bone.^29^ If cortical bone is replaced by cancellous bone, the resistance to bending is decreased, and the bending moment required to produce a certain strain is decreased. This should imply that if there exists a means to reduce the thickness of the cortical bone and to replace this by cancellous bone, this should be instrumental in maintaining the buccal-lingual dimensions of the mandible ridge. Furthermore, the modulus of elasticity of cancellous bone varies widely, which means that if there exists a means to get cancellous bone of a low modulus of elasticity, this should also be instrumental in maintaining the buccolingual dimensions of the mandible ridge.

## Discussion

Physiology is the science about the physical and chemical functions of a living body. This article deals with the physical aspect of bone physiology. The language of physics is mathematics. It would have been natural to express the line of arguments of this study in a strictly mathematical language. However, in the interest of readability, the message has been worded in a qualitative manner.

The above analysis shows that the changes in the dimensions of the alveolar ridge observed after tooth extractions and after placement of implants in fresh extraction sockets appear to be a natural consequence of the biologic laws according to which the body is designed. In the evolution, in the struggle for life, it has been important not to be too heavy. For this reason, nature economizes with bone; it gets rid of bone that is not sufficiently used by which the daily stress/strain stimulus seems to be the measure of use applied.^23^

The above analysis was made on the mandible since the mandible exhibits many similarities with a common engineering structure – a curved beam. It is however suggested that the same line of arguments can be applied on the maxilla with its more complicated anatomy. Like the mandible, the maxilla, in a mechanical sense, behaves as if the space occupied by the periodontal ligament and the tooth was empty. When, after tooth extraction, this space is occupied by bone or by an implant and bone, the stiffness of the maxilla will be increased. With unchanged loads, increased stiffness implies reduced strains. The strain stimulus needed to maintain bone mass is no longer reached. The biologic response to this is to remove bone, which is preferentially performed at the external bone envelope. The dimensions of the alveolar ridge will be reduced.

Freeze-dried bone allograft in combination with a membrane was found to improve the ridge dimensions in patients after 6 months,^12^ and in dogs grafting with Bio-Oss collagen in extraction sockets was found to improve the ridge dimensions, also after 6 months.^13^ It was suggested above that a theoretical possibility to maintain the ridge dimensions after tooth extraction is to have the extraction socket filled with bone of a low modulus of elasticity. It can be speculated that the freeze-dried allograft and the Bio-Oss collagen achieved that. A question that immediately presents itself is ‘what will happen in the long run?’ Will the modulus of elasticity of the bone filling the extraction socket increase with time? Grafting with autologous bone did not improve the ridge dimensions after 6 months.^14^

A consequence of tooth extraction is alveolar ridge resorption.^2,36^ The placement of implants in fresh extraction sockets has failed to prevent this bone modelling process.^8^ The present analysis shows that this reduction of the dimensions of the alveolar ridge after tooth extraction seems to be a natural consequence of well-known physiological laws. After healing of the extraction socket, the strain stimulus needed to maintain bone mass is no longer reached. The bone resorption is normally larger at the buccal aspect of the ridge than at the lingual aspect.^7,37,38^ In animal and clinical studies, the vertical component of the bone loss has been more pronounced at the buccal aspect.^8,38^ A consequence of a greater vertical bone loss buccally than lingually is a ridge that is sloped in the lingual-buccal direction. In cases with such a sloped alveolar ridge anatomy, the placement of a standard implant might not be optimal. The placement of the implant in level with the lingual bone margin may result in compromised aesthetics. If the implant instead is placed in level with the buccal bone margin, the lingual marginal bone is at risk to be resorbed due to insufficient strain stimulus. In a clinical study, Fiorellini et al.^39^ used an implant with a sloped marginal contour in cases where the patient presented with an alveolar crest that was sloped in the lingual to buccal direction. Both the mean buccal marginal bone level change and the mean lingual marginal bone level change after 16 weeks amounted to −0.2 mm. Thus, the installation of an implant with a sloped marginal contour may be a treatment option in cases where the alveolar ridge is sloped in lingual to buccal direction.

## Conclusion

The reduction of the buccolingual as well as the apicocoronal dimension of the alveolar ridge, commonly observed after tooth extraction, can be explained by the physiological law, according to which the maintenance of the bone anatomy requires a certain daily stress/strain stimulus.
